# Barriers to domestic violence disclosure in healthcare settings: a scoping review of victim and provider perspectives

**DOI:** 10.1186/s12913-025-13709-2

**Published:** 2026-01-30

**Authors:** Tamana Barakati, Manu R. Mathur, Manas Dave, Mohammad I. Farook, Simon Holmes, Ali Golkari, Ania Korszun, Mohammad S. A. Alshammari, Paul Coulthard

**Affiliations:** 1 Faculty of Medicine and Dentistry, Queen Mary University of London, Office 3, 4th Floor, Institute of Dentistry, Turner Street, Whitechapel, London, E1 2AD UK; 2 Bloomsbury Trust, London, UK

**Keywords:** Domestic violence, Intimate partner violence, Barriers, Disclosure, Healthcare services, Health services accessibility, Cultural competency

## Abstract

**Background:**

Domestic violence (DV) is a global public health issue with far-reaching physical, psychological, and social consequences. Although prior reviews have identified barriers to DV disclosure in healthcare settings, these have predominantly focused on female victims in Western contexts. This scoping review builds on the work of Heron and Eisma (2021) by including male victims, along with females, and studies from Asian countries, along with western countries, offering a more inclusive and culturally diverse understanding of disclosure barriers.

**Methods:**

A thorough search of four databases: PubMed, Scopus, Embase, and the Cochrane Database, was conducted to identify relevant studies published between January 2018 and April 2023. Studies were included if they examined barriers to DV disclosure in healthcare settings from the perspectives of either victims or healthcare professionals (HCPs). Title and abstract screening, full-text review, and data extraction were performed independently by two reviewers. A thematic analysis was conducted to synthesise victim- and HCP-related barriers.

**Results:**

Fifteen studies met the inclusion criteria. Victim-reported barriers included fear of retaliation, social stigma, low self-esteem, mental health challenges, and lack of privacy during healthcare encounters. Male victims highlighted societal disbelief and stigma around male victimhood. In Asian countries, cultural norms around family honour and obedience were particularly influential in discouraging disclosure. Practical barriers, such as the presence of abusers and limited access to services, were common in both high-income and low- and middle-income settings. HCP-reported barriers included inadequate training, absence of standardised protocols, time constraints, and a lack of culturally sensitive tools.

**Conclusion:**

This review identifies complex, context-specific barriers to DV disclosure, especially for male victims and individuals in non-Western healthcare systems. Addressing these barriers requires gender-sensitive training, culturally appropriate interventions, and systemic improvements to healthcare delivery. These findings call for inclusive, evidence-based strategies to support disclosure and improve care for all DV survivors in healthcare settings.

## Background

Domestic violence (DV) is a pervasive public health concern and a severe violation of human rights, with significant consequences for physical, psychological, and social well-being worldwide [1]. In the United Kingdom, the Home Office defines DV as “any incident or pattern of incidents of controlling, coercive or threatening behaviour, violence or abuse between those aged 16 or over who are, or have been, intimate partners or family members, regardless of gender or sexuality” [2, 3]. This broad definition encompasses physical, emotional, sexual, and economic abuse, as well as technology-facilitated abuse and acts linked to so-called “honour”-based violence [4]. Terms such as *intimate partner violence (IPV)*, *domestic abuse*, and *domestic violence and abuse (DVA)* are often used interchangeably in the literature [5].

The health impacts of DV are wide-ranging and multifactorial. Victims may experience both acute injuries—such as fractures, contusions, and head trauma—and chronic health conditions, including gynaecological disorders, gastrointestinal disturbances, and persistent pain syndromes [6–8]. The psychological toll is equally severe, with elevated rates of depression, anxiety, and post-traumatic stress disorder (PTSD) documented among DV survivors [9]. Children exposed to DV are at heightened risk for long-term emotional, cognitive, and behavioural difficulties, with potential effects that extend into adulthood [8].

Globally, DV disproportionately affects women. According to the World Health Organization (WHO), intimate partners are responsible for between 28% and 50% of all female homicides [10]. Approximately 26–28% of women aged 20–44 years report experiencing DV, and 10–16% report having been subjected to sexual violence by a current or former partner [10, 11]. Although men report significantly lower rates of victimisation, male experiences of DV remain underrepresented and under-researched [1].

Despite the high prevalence and serious health implications, DV remains markedly underreported. Victims often face numerous personal and systemic barriers to disclosure [12]. These include fear of retaliation, shame, social stigma, and concerns about losing child custody or being judged by healthcare professionals (HCPs) [13]. Some victims may normalise abusive behaviours or fear they will not be believed, further inhibiting disclosure [14]. Practical constraints—such as limited consultation time, lack of privacy, and insufficient HCP training to recognise and respond to DV—further impede the process [1, 15–17].

Healthcare professionals also encounter challenges in identifying and supporting DV victims. Barriers include limited training, absence of culturally sensitive guidelines, discomfort in raising the topic, and systemic issues such as resource limitations and time pressures. The presence of the perpetrator during consultations may also prevent safe inquiry or intervention [1, 15–17]. A prior systematic review by Heron and Eisma [14]. documented many of these barriers but focused primarily on female victims in Western healthcare settings, omitting male perspectives and data from non-Western contexts.

This scoping review aims to address these gaps by including all studies that explore barriers to DV disclosure among both male and female victims and adding Asian countries to the context. It also incorporates findings related to victim counselling and disclosure outcomes—areas previously underexplored. By broadening the scope, the review offers a more inclusive understanding of the structural, cultural, and interpersonal factors that hinder DV disclosure in healthcare settings globally. Specifically, it seeks to address the following research questions:

1. What are the barriers that prevent victims from disclosing DV in healthcare settings?
2. What challenges do healthcare professionals face in facilitating such disclosures?

## Methods

### Review strategy

This scoping review aimed to synthesise evidence on the barriers to DV disclosure in healthcare settings, as reported by both victims and healthcare professionals (HCPs). It builds on the work of Heron and Eisma [14, 16] by extending the scope to include: (1) studies involving male victims, an underrepresented group in previous reviews; and (2) outcomes related to DV counselling and disclosure, in addition to previously established barriers. The review followed the Preferred Reporting Items and PRISMA-ScR guideline for scoping reviews [18].

### Search strategy

This scoping review employed a targeted literature search strategy, developed in collaboration with the Library Services team at Queen Mary University of London. The following electronic databases were searched: Medline, Scopus, Embase, and the Cochrane Database of Systematic Reviews.

The search covered studies published between January 2018 and April 2023, selected to avoid overlap with the time frame of the Heron and Eisma review (1996–2018) [11] and to ensure the inclusion of recent literature.

Search terms combined Medical Subject Headings (MeSH) and keywords, including:

*“Domestic Violence”*,* “Intimate Partner Violence”*,* “Domestic Abuse”*,* “Barriers”*,* “Communication Barriers”*,* “Disclosure”*,* “Reporting”*,* “Healthcare”*, and *“Health Services”*.

The strategy was refined iteratively to enhance sensitivity and specificity. Where appropriate, technical terms were supplemented or replaced with lay terms, and longer phrases were broken into simpler search strings. A manual search of reference lists from included studies was performed to identify additional eligible studies.

### Search filters

- Publication date: January 2018 to April 2023.
- Population: Studies exploring barriers to DV disclosure from the perspectives of victims or healthcare professionals.
- Access: Full-text availability via print or electronic resources (free or institutional access).
- Language: Articles published in English.
- Study type: Primary or secondary research (qualitative, quantitative, or mixed methods).

### Title and abstract screening

The search results from all databases were imported to Endnote Desktop version X20 (Clarivate, Philadelphia, PA, USA). Duplicates were first removed using EndNote’s automatic duplicate detection tool. Remaining records were manually checked by title and author to identify any duplicates that were not detected by the software.

Screening and selection were conducted independently by two reviewers (TB and MM). Disagreements were resolved through discussion, with arbitration by a third reviewer (PC) when necessary. Articles lacking full-text access were excluded without contacting authors for copies.

### Quality screening

The quality of the included studies was appraised using the Critical Appraisal Skills Programme (CASP) checklists, with the appropriate version applied according to each study design. Studies receiving a full score were classified as *high quality*, those missing one criterion were considered *medium quality*, and those missing two or more criteria were rated as *low quality*. Studies rated as low quality were excluded from the final synthesis. For studies rated as medium quality, inclusion decisions were made through group discussion among the authors, based on the importance of the omitted criterion and its relevance to the overall methodological rigor required for this scoping review.

### Full text assessment

Those studies that remained in the study after screening, went under full text assessment. Those that were found not directly related were excluded. The rest went under final review and data extraction.

### Data extraction and synthesis

Data extraction was performed independently by two reviewers (TB and MM) using a standardised template, focusing on:

1. Study characteristics (e.g. year, country, design).
2. Participant demographics.
3. Identified barriers to DV disclosure.

Barriers were grouped into two overarching themes:

- Victim-related barriers.
- Healthcare professional-related barriers.

Due to the methodological and contextual heterogeneity among studies (as anticipated based on prior reviews), a descriptive thematic analysis was undertaken. This qualitative synthesis approach enabled the identification of cross-cutting themes and contextual nuances without conducting a meta-analysis. Data were cross-validated between reviewers to ensure consistency and reliability.

## Results

### Study selection

**Fig. 1:**
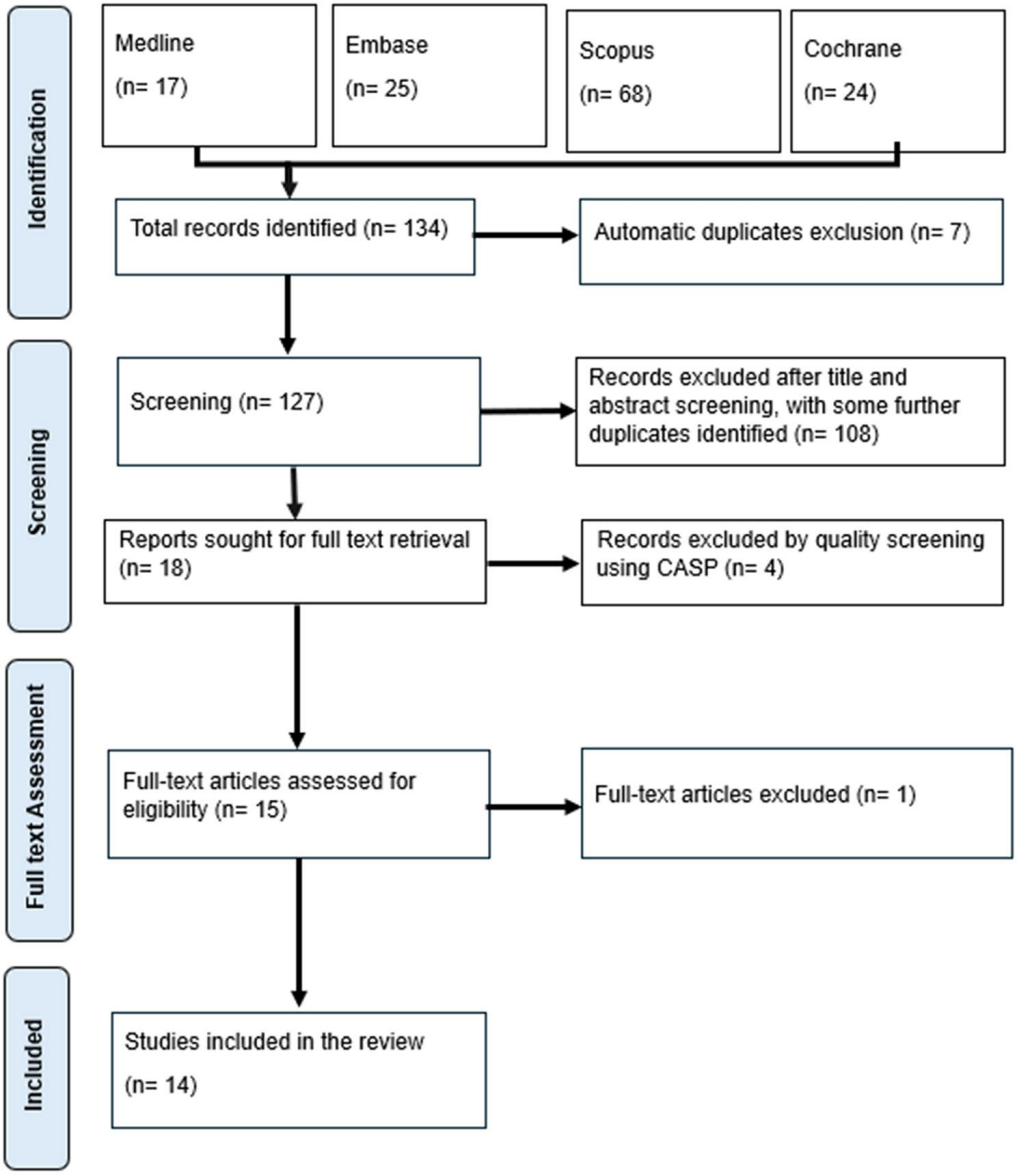
PRISMA flow diagram illustrating the study selection process for this scoping review

The initial search yielded 134 records. After automatic removal of duplicates (*n* = 7), 127 studies remained. Of these, a total of 112 were excluded in the next phases: 108 studies were excluded during screening of the relevance of title and abstract (some more duplicates were identified in this phase), four were excluded during quality appraisal using CASP tool, and one study was removed by full text assessment for eligibility. The remaining 14 studies underwent full-text review and were all included in the final synthesis. The screening process is summarised in the PRISMA flow diagram (Fig. 1).

### Characteristics of included studies

The 14 included studies comprised a combination of quantitative, qualitative, and mixed methods designs. Ten studies were conducted in high-income countries, including the United States (*n* = 4), the United Kingdom (*n* = 3), Australia (*n* = 2) and Hong Kong (*n* = 1). Four additional studies were conducted in low- and middle-income countries (LMICs) of Uganda, Sri Lanka, Iran, and India (*n* = 1 each).

Collectively, the studies captured the perspectives of 2,311 female participants and 341 male participants across six countries on three continents. While all studies explored barriers to DV disclosure from the victim’s perspective, nine also examined challenges faced by healthcare professionals (HCPs) in facilitating disclosure [1, 14, 16, 19–24]. Table 1 presents a summary of study designs, geographic distribution, populations studied, and key findings.

**Table 1:** Summary of study characteristics and key findings from the included studies

Author Year	Country	Study design	Participants	Identified barriers of disclosing DV to HCPs
Sun, Lam [19] 2019	Hong Kong	Qualitative and quantitative	*N* = 504	- Lack of guidelines and support. - HCPs limited skills and time. - Victims’ reluctance to disclose.
Vranda, Kumar [25] 2019	India	Qualitative (semi-structured interviews)	*N* = 100 Female	- Fear of threats and more violence from husband/mother-in-law. - Embarrassment/shame. - Fear of re-traumatisation. - Fear of confinement and husband will not bring for follow-up for treatment. - Fear that nobody would believe them. - Had no physical injuries due to violence and it was only emotional violence. - Presence of partner and lack of privacy.
Karimi-Shahanjarini, Shakibazadeh [20] 2019	Iran	systematic review of qualitative studies	66 studies (69 papers)	- Victims perceived nurses were more easily accessible than doctors.
Huntley, Potter [1] 2019	United Kingdom	Systematic review and qualitative evidence synthesis	Not mentioned	- Fear of disclosure of DVA. - HCP related factors.
Hasselle, Howell [26] 2020	United States of America	Qualitative (interview/ feedback sessions)	*N* = 9 Female	- Mental health barriers (e.g., depressive symptoms). - Pregnancy and health-related barriers (e.g., physical health symptoms). - Partner-related barriers (e.g., direct interference or demoralisation from a violent partner). - Practical barriers (e.g., transportation, lack of compensation, and childcare) - Cultural barriers (e.g., normalisation of IPV and societal stigma). - Perceived systemic barriers (e.g., fear or mistrust of helping systems and lack of available resources.
Portnoy, Colon [21] 2020	United States of America	Qualitative (interviews + focus groups)	*N* = 10 Patient + 29 providers	- Limited time in the session. - Scope of practice and role of HCP. - Providers’ own reactions to IPV and expectations for screening.
Wright, Anderson [22] 2021	United States of America	Systematic review	Not mentioned	- Cultural and familial beliefs. - Lack of healthcare response. - Lack of resources or knowledge. - Social stigma and norms. - Fear.
Creedy, Baird [27] 2021	Australia	Cross-sectional survey	*N* = 144 Female *N* = 23 Male	- Presence of partner. - Language barriers.
Heron and Eisma [14] 2021	United Kingdom	systematic review of qualitative research	34 studies with a total 781 Female and 2 Male participants	- Fear of the consequences of disclosing DV. - Victims feared being judged/ negatively evaluated by either the HCP or their environment, family, friends, neighbours, acquaintances. - Victims communicated that fear of their abuser further prevented them from disclosing. - A lack of a positive relationship with the HCP. - Personal barriers such as a low self-esteem, feelings of shame, embarrassment, guilt and powerlessness.
Heron, Eisma [16] 2022	United Kingdom	Qualitative study (exploratory, interview-based)	*N* = 22	- Fear. - Embarrassment or shame; self-blame. - Partner’s physical presence. - Partner’s controlling behaviour. - Partner’s manipulation of health care professionals. - Inappropriate setting. - Insufficient time with HCP.
Anguzu, Cassidy [23] 2022	Uganda	Qualitative (in-depth interviews)	*N* = 28	- Staffing shortages - Inadequate physical space. - Lack of comprehensive IPV training. - Inadequate IPV screening knowledge. - Need for brief IPV screening tools. - HCP misperceptions. - HCP initiated probing. - Rapport building. - Limited staffing and space resources. - Lack of comprehensive gender-based violence (GBV) training.
Traynor, Schmidt [28] 2023	United States of America	secondary data analysis of randomised control trials using latent profile analysis	*N* = 801	- Housing instability - Recent incarceration - Unemployment - Low income - Language barriers - Lack of transportation
Silva, Agampodi [24] 2022	Sri Lanka	Qualitative (Interview-based)	*N* = 20 Female	- Women’s lack of knowledge and perceptions about the role of HCPs. - Lack of confidence in HCPs. - Fear of repercussions. - Personal attitudes towards DV. - Their love and loyalty towards the perpetrator.
Usanov, Keedle [29] 2023	Australia	Qualitative (semi-structured interviews)	*N* = 12	- Physical, financial and relational consequences: ‘Fear of the ramifications’. - Self-blame and rationalisation of abuse: ‘It’s my fault’. - Ashamed to disclose: ‘Shame, stigma, not wanting to tell anyone’ - Caring for both women who have experienced domestic violence and their perpetrators: ‘Conflicts of interest and whatnot’. - Not knowing what to do: ‘Fear of opening a can of worms’. - Knowledge and awareness deficits: ‘Education’s a huge thing’. - Someone else does it: ‘Screening happens in the hospital’ EXOSYSTEM Organisational structures not conducive to screening. - Inhibited by time constraints: ‘Under a lot of time pressure’. - Lacking physical resources and emotional support: ‘We do not seem to have much support’. - Not at the forefront: ‘Off your radar’. - Ethnic considerations: ‘Culture has a lot to play with things’ socioeconomic assumptions.

Thematic analysis of the 14 included studies revealed a range of barriers to DV disclosure, which were categorised into two major groups: barriers experienced by victims and barriers encountered by HCPs. The themes below summarise the key findings within each category, with illustrative examples drawn from diverse geographic and cultural settings.

### Key themes and findings

### A. Barriers to disclosure experienced by victims

#### A.1. Fear

Fear appeared as a dominant theme across all studies and encompassed various concerns, including fear of retaliation, losing custody of children, and being judged by HCPs or society. For instance, victims in the United Kingdom and Australia highlighted fear of abusers’ manipulation as a key deterrent [16, 29]. Studies from India and Sri Lanka further revealed that women feared threats of further violence or social ostracization if they disclosed abuse [24, 25]. Fear was also a significant barrier for male victims, who expressed concerns about being disbelieved or ridiculed due to societal perceptions that men cannot be victims of DV [1].

#### A.2. Controlling behaviour

Abusers’ controlling behaviours, such as monitoring victims’ movements, attending healthcare appointments, and restricting access to social networks, were reported as significant barriers to disclosure. This was evident in studies from the United Kingdom and Uganda, where victims highlighted how their abusers’ presence inhibited open communication with HCPs [14, 23].

#### A.3. Concerns about family disruption

Some victims reported fear of being forced to leave their homes if they disclosed the abuse, particularly when financially or socially dependent on the abuser. In several studies, particularly from high-income countries, victims expressed anxiety over losing custody of their children if social services were involved [22, 26]. This concern was exacerbated in patriarchal societies like India, where victims feared that disclosure would disrupt family structures (26).

#### A.4. Cultural and societal norms

Cultural expectations of loyalty and honour often deterred victims from seeking help. In Sri Lanka and Iran, for example, women reported feeling obligated to remain silent to preserve family reputation [20, 24]. Such cultural norms also influenced male victims, who felt pressure to conform to traditional gender roles, which discouraged them from disclosing abuse [1].

#### A.5. Practical constraints

Practical issues, such as lack of privacy during healthcare appointments, were consistently identified as barriers. Victims in Australia, the United States, and Uganda reported that the presence of their abuser during consultations prevented disclosure [23, 27]. Limited transportation and childcare further constrained victims in low-resource settings, as highlighted in Uganda and the United States [23, 28].

#### A.6. Mental and physical health morbidity

Mental health conditions, including depression and anxiety, were frequently associated with delayed disclosure. Victims from the United States and Australia noted that these conditions reduced their capacity to seek help [26, 29]. Similarly, victims in Hong Kong prioritised addressing immediate physical health issues over discussing abuse [19].

#### A.7. Lack of knowledge

Victims’ lack of awareness about available resources and the role of HCPs in addressing DV was another significant barrier. In Sri Lanka and Iran, women expressed doubts about HCPs’ ability to provide meaningful assistance [20, 24]. Male victims, similarly, reported being unaware of services tailored to their needs, reflecting a broader gap in victim-specific education [1].

#### A.8. Emotional and psychological burdens

Several studies highlighted internal psychological barriers that discouraged victims from disclosing abuse. Feelings of self-blame, low self-esteem, and guilt were reported, particularly among female victims who internalised societal messages about family loyalty and personal responsibility. In Sri Lanka and Iran, women described feeling responsible for the abuse or worried that disclosure would bring shame upon their families [20, 24]. Similarly, low self-worth led some victims to believe they were undeserving of help or that the abuse was justified. These emotional burdens compounded other structural and social barriers, further inhibiting the likelihood of seeking support from healthcare professionals.

### B. Barriers to facilitating disclosure faced by healthcare professionals

#### B.1. Knowledge and training deficits

HCPs universally reported insufficient training on identifying and managing DV. In Uganda and Hong Kong for example, a lack of comprehensive gender-based violence (GBV) training led to missed opportunities for intervention [19, 23]. In Iran, nurses were perceived as more accessible than doctors, yet they too lacked formal education on IPV screening [20]. Male victims highlighted the lack of provider knowledge about male-specific DV experiences [1].

#### B.2. Structural and resource constraints

Time constraints during consultations and insufficient private spaces were frequently cited as barriers. HCPs in Uganda and the United States reported that overcrowded facilities and limited staff capacity hindered effective engagement with victims [21, 23]. These challenges were particularly pronounced in LMICs, where resource limitations further compounded the issue.

#### B.3. Negative attitudes and perceptions

Victims perceived HCPs as dismissive or judgemental, which discouraged disclosure. In Sri Lanka and Australia, HCPs themselves admitted discomfort discussing DV, often deferring responsibility to external services [24, 29]. Male victims, in particular, highlighted the stigma associated with male victimhood, noting that HCPs often questioned the legitimacy of their claims [1].

#### B.4. Lack of guidelines and standardised protocols

HCPs consistently identified the absence of clear guidelines as a barrier to addressing DV. In Hong Kong and the United States, providers reported uncertainty about how to respond to disclosures due to a lack of institutional protocols [19, 21].

### Synthesis of findings

Table 2 summarises the identified barriers under the two main categories. Victims commonly reported fear, stigma, and lack of knowledge, while HCPs cited training deficits, unclear protocols, and limited privacy during consultations.

**Table 2:** Thematic synthesis of barriers to domestic violence disclosure reported by victims and healthcare professionals

Barriers to disclosure of DV for victims	Barriers to disclosure of DV for healthcare practitioners
• Fear • Controlling behaviour • Concerns for losing their children • Concerns for leaving their home • Betraying the abuser (often an intimate partner or family member) • Embarrassment • Self-blame • Low self-esteem • Feeling guilt • Mental and physical health morbidity • Depression and anxiety • Lack of knowledge	• Lack of response • Lack of positive relationship between victim and HCP • Lack of knowledge • Lack of awareness • Lack of education • Lack of guidelines to support victims • Limited skills and time • Presence of abuser with the victim at healthcare appointments • Privacy of victim whereby the HCP is not able to record the disclosed DV in clinical records

## Discussion

By building on the work of Heron and Eisma [14], this review addresses critical gaps in the literature, focusing on previously underrepresented groups and geographies. The findings underscore the multifaceted nature of barriers to DV disclosure, which are shaped by psychological, cultural, and systemic factors. While many of the barriers identified in this review align with Heron and Eisma [14] findings, the inclusion of male victims and Asian contexts reveals unique insights and highlights substantial variations.

The thematic analysis revealed distinct and overlapping challenges faced by both victims and healthcare professionals. These findings are discussed below in relation to previous literature and contextual nuances.

### Victim-related barriers to disclosure

Although Heron and Eisma [14] identified fear as the principal barrier, this review shows that its expression diverges across different contexts. In Asian countries such as India and Sri Lanka, cultural norms that emphasise family honour amplify victims’ fear of social judgment and ostracism [24, 25]. This contrasts with Western environments, where fear is more commonly linked to retaliation by the abuser and legal repercussions [16]. Moreover, male victims in both Western and Asian contexts reported fear of disbelief and ridicule, reflecting entrenched gender expectations that discourage men from seeking assistance [1]. Community-level interventions and inclusive healthcare policies are needed to reduce fear and hesitation among male victims of DVA. Public awareness campaigns that challenge gender stereotypes can help normalize help-seeking behaviors. Strengthening confidentiality, trust, and inclusivity in service provision would further support men in accessing appropriate care.

Stigma exerts a particularly profound effect in Asian societies. For example, women in Sri Lanka described guilt and shame at the prospect of leaving their abuser, shaped by cultural obligations of loyalty [24]. By contrast, male victims in Western societies emphasised stigma related to masculinity and the erroneous notion that men cannot be victims of abuse [1]. Collectively, these findings warrant the necessity of culturally tailored interventions by specifying community-based education and awareness programs that challenge gender norms, training for healthcare and social service providers to ensure sensitive and inclusive responses, and the development of confidential, gender-responsive support services. These interventions can better reflect cultural contexts and effectively meet the diverse needs of different victim populations.

Practical barriers, such as an abuser’s presence or insufficient privacy, remain consistent with Heron’s findings, but this review highlights further complications in low-resource settings. In Uganda and Hong Kong, for example, limited transport and childcare posed serious hurdles for victims [19, 23]. The overlap of practical and cultural barriers in Asia was also evident, as victims described rigid familial constraints that limited healthcare access without the abuser’s knowledge [25].

One of the most substantial contributions of this review is the inclusion of male victim experiences. Male victims across both Western and Asian countries reported feeling excluded from existing support structures, which are often designed with female victims in mind [1]. Additionally, men expressed reluctance to disclose abuse due to fears of being perceived as weak or responsible for their victimisation. These findings underscore the need for gender-sensitive training for healthcare professionals (HCPs) and the development of resources specifically tailored to male victims.

### Healthcare professional-related barriers

While Heron and Eisma [14] identified inadequate training as a barrier, this review underscores the critical need for context-specific training. HCPs in Uganda and Hong Kong highlighted the absence of culturally appropriate IPV screening tools and protocols, which hinder their ability to address DV effectively [19, 23]. Moreover, studies from Western countries revealed a lack of training on addressing male-specific DV experiences, further limiting HCPs’ capacity to support diverse victim populations [1].

The review identifies significant disparities in healthcare infrastructure between high-income and low- and middle-income countries (LMICs). In Uganda, for instance, HCPs cited staffing shortages and inadequate physical space as critical barriers to facilitating DV disclosure [23]. These findings underscore the compounded structural challenges faced by HCPs in resource-limited settings, which were not extensively addressed in Heron’s review.

This review expands on Heron’s findings by highlighting the role of cultural insensitivity among HCPs. In Sri Lanka and Iran, victims reported feeling dismissed or misunderstood by HCPs who lacked awareness of the cultural context of DV [20, 24]. Similarly, male victims noted that HCPs often trivialised their experiences, reinforcing societal stereotypes about masculinity and victimhood [1]. These findings indicate that culturally sensitive training should address biases and improve HCP–victim interactions. For male victims, this includes recognizing stigma, validating experiences, and ensuring confidentiality. Training should also promote gender-inclusive services and provide case examples to enhance empathetic, appropriate responses [30, 31].

While this review found that many healthcare professionals reported a lack of clear guidelines for responding to DV disclosures, it is important to acknowledge that internationally recognised resources do exist. For example, WHO has developed a clinical handbook for healthcare providers on how to respond to intimate partner violence and sexual violence, which includes the widely endorsed LIVES approach (*Listen*,* Inquire*,* Validate*,* Enhance safety*,* Support*) [32]. This model has been adopted in various settings. For instance, in Australia, the Royal Australian College of General Practitioners (RACGP) incorporated LIVES into national training and professional guidance for general practitioners and healthcare institutions [33]. However, the persistence of reported guideline-related barriers in studies from both high-income countries and LMICs suggests that such resources are often not disseminated effectively, not locally adapted, or not integrated into routine practice. This highlights the need for improved training, contextualisation of existing global frameworks, and better institutional support to ensure that healthcare professionals are both aware of and equipped to implement these guidelines in diverse care environments.

### Strengths and contributions

The study benefited from a well-designed and targeted search strategy to capture only the most relevant literature, alongside a rigorous quality screening using CASP tools to ensure methodological robustness. This review also makes several unique contributions to the literature:

1. **Male Victims**: By including male victims, this review highlights gender-specific barriers that have been largely overlooked in prior research. These findings underscore the need for gender-sensitive policies and interventions.
2. **Asian Contexts**: The inclusion of studies from four Asian countries provides valuable insights into the cultural and systemic factors that influence DV disclosure. The intersection of cultural norms, resource limitations, and societal stigma in these settings highlights the need for context-specific approaches.
3. **Intersectionality**: The findings illustrate how intersecting factors, such as gender, culture, and resource availability, shape barriers to DV disclosure, offering a more nuanced understanding than previous reviews.

### Limitations

Despite its strengths, this review has some limitations. The exclusion of non-English studies may have limited the scope of findings, particularly from non-Western contexts where DV is underreported. Additionally, the reliance on published, peer-reviewed literature excludes grey literature, which may contain valuable insights. The synthesis of HCP-related barriers across diverse professional roles (e.g., nurses, physicians, social workers) limits the ability to draw role-specific conclusions [34]. Furthermore, while a quality appraisal was conducted using the CASP checklist, the absence of detailed risk of bias scoring is acknowledged as a limitation, as it may influence the interpretability of findings.

### Implications for research, practice, and policy

#### Research

Future research efforts should emphasise:

- Examining barriers faced by diverse groups (e.g., ethnic minorities, LGBTQ + individuals, and victims in LMICs)[35, 36].
- Assessing the efficacy of culturally adapted interventions aimed at improving DV disclosure [37].
- Developing standardised measures for DV prevalence and disclosure [38].

### Practice

Healthcare providers would benefit from thorough training to:

- Recognise potential DV victims using validated screening tools [39, 40].
- Establish safe, confidential conditions for disclosure [41, 42].
- Build a rapport and provide clear referral pathways to support services.

In addition, patient education should be prioritised, incorporating accessible materials that raise awareness of DV and outline available support resources [43].

## Policy

Policy measures should include:

- Mandating IPV screening in high-risk healthcare environments [44].
- Allocating funding for training programmes and the enhancement of support services.
- Encouraging collaboration between healthcare systems and social service agencies to ensure a coordinated response to DV [45].

## Conclusion

This scoping review provides an inclusive synthesis of barriers to domestic violence (DV) disclosure in healthcare settings. By incorporating the perspectives of male victims and including studies from Asian countries, it extends the findings of prior reviews and highlights the diverse, intersectional challenges that shape disclosure practices.

As summarised in Table 2, victims commonly face psychological, cultural, and practical barriers such as fear, stigma, controlling partners, and lack of privacy, while healthcare professionals (HCPs) encounter challenges related to inadequate training, absence of clear guidelines, and systemic limitations. These barriers are often compounded in low-resource or culturally conservative settings, underscoring the need for context-specific, gender-sensitive, and culturally informed interventions.

To improve outcomes, healthcare systems must implement comprehensive training for HCPs, establish confidential environments for patient interaction, and develop standardised protocols that recognise the diverse realities of DV victims. Addressing these barriers is essential to ensuring that all individuals (regardless of gender, culture, or geography) receive the support they need within healthcare settings.

## Data Availability

All data generated or analysed during this study are included in this published article. Supplementary information, including files related to search strategy and results, is available from corresponding author on reasonable request.
